# Interactions between Loci Contributing to Antimicrobial Resistance and Virulence in Neisseria gonorrhoeae

**DOI:** 10.1128/mbio.00412-22

**Published:** 2022-04-14

**Authors:** Tatum D. Mortimer

**Affiliations:** a Department of Immunology and Infectious Diseases, Harvard T.H. Chan School of Public Health, Boston, Massachusetts, USA

**Keywords:** *Neisseria gonorrhoeae*, antibiotic resistance, gonorrhea, virulence factors

## Abstract

In a recent mBio article, Ayala et al. (mBio 13:e00276-22, 2022, https://doi.org/10.1128/mbio.00276-22) identified a single nucleotide variant in the repressor *gdhR* in Neisseria gonorrhoeae that reduces binding to the promoter of the virulence factor *lctP* and thereby increases its expression. The allele (*gdhR6*) frequently co-occurs with mutations in the *mtr* operon promoter that reduce expression of another repressor, *mtrR*, resulting in overexpression of the efflux pump-encoding *mtrCDE* and increased antimicrobial resistance. Because *mtrR* also represses *gdhR*, a decline in *mtrR* would decrease expression of *lctP.* Hypothesizing that *gdhR6* arose to circumvent the impact of *mtrR* promoter mutations on *lctP* expression, the authors analyzed these loci in genomes of N. gonorrhoeae isolates from the preantibiotic era. Surprisingly, they found isolates with *gdhR6* prior to selection for *mtrR* resistance-associated alleles. These results suggest that independent and perhaps interacting pressures have influenced the co-occurrence of these alleles.

## COMMENTARY

Infection with Neisseria gonorrhoeae has been increasing in incidence over the past decade, and treatment options are limited due to growing antimicrobial resistance (1). The gonococcal population is, in part, structured by the presence of resistance-associated alleles (2), but interactions between resistance loci and other genetic variation remain largely unexplored. One well-characterized mechanism of resistance in N. gonorrhoeae is upregulation of the multidrug efflux pump MtrCDE, encoded by *mtrCDE*. While many variants can contribute to increased *mtrCDE* expression, including those that impact the function of the *mtrR* repressor or its binding to the *mtr* promoter, one of the most common mutations observed in clinical isolates is a single adenine deletion in the promoter. This allele (*mtrR-*P A-del) simultaneously decreases expression of *mtrR* and increases *mtrCDE* expression (3). The impacts of resistance-associated mutations in the *mtr* operon on N. gonorrhoeae fitness and virulence are complex. Expression of *mtrCDE* has been shown to confer a fitness benefit in the mouse colonization model (4); however, loss-of-function mutations in *mtrC* are also overrepresented in isolates from cervical infections, suggesting that efflux pump activity is costly in some environments (5). MtrR also regulates the expression of several non-efflux pump-related genes, so mutations in *mtrR* or its promoter could have effects on gonococcal biology beyond antimicrobial resistance.

**FIG 1:**
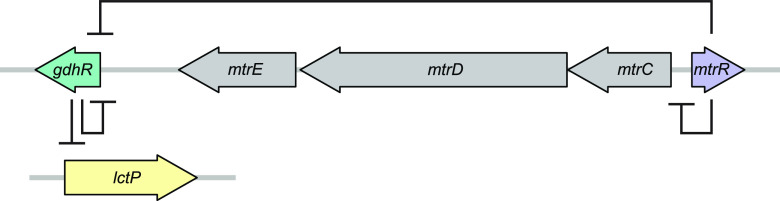
Regulation of *lctP* by *mtrR* and *gdhR. mtrR* (purple) encodes the repressor of the efflux pump-encoding operon, *mtrCDE* (gray) (3). MtrR also represses *gdhR* (green), which encodes a GntR-like DNA-binding protein (11). GdhR represses its own expression (11) and *lctP* (yellow), a gene encoding the lactate permease (12).

In a recently published article in mBio, Ayala et al. (6) focused on *gdhR*, a repressor of the virulence factor *lctP*, which is itself repressed by *mtrR* (Fig. 1). *lctP* encodes the l-lactate permease, and its expression impacts survival in the mouse model and serum resistance through lipopolysaccharide sialylation (7). Since mutations that downregulate *mtrR*, like *mtrR-*P A-del, would also affect the expression of *lctP* via *gdhR*, the authors hypothesized that N. gonorrhoeae encodes mutations in *gdhR* to circumvent this impact. Using a combination of molecular genetics, structural biology, and population genomics, they studied the role of genetic diversity in *gdhR* on *lctP* expression and the association between alleles in *gdhR*, the *mtr* operon, and antimicrobial resistance.

The majority of *in vitro* and *in vivo* studies of N. gonorrhoeae are performed with one or a few common laboratory strains. The authors examined the sequence of *gdhR* in five commonly used N. gonorrhoeae strains and identified a single nucleotide variant in codon 6 corresponding to a proline-to-serine change (*gdhR6*) in strain MS11. To define the potential clinical relevance of *gdhR* alleles beyond laboratory strains, mutations in *gdhR* were also identified in a data set of 300 isolates collected in 2017 to 2018 as part of the U.S. Centers for Disease Control and Prevention’s Gonococcal Isolate Surveillance Program (GISP) (8). *gdhR6* was the most common non-wild-type allele and was present in 19.7% of gonococcal genomes in the GISP data set.

Expression of *lctP* is higher in MS11 than in other laboratory strains. The authors show that this difference is attributable to the *gdhR6* allele: *lctP* expression is higher in isogenic mutants encoding *gdhR6* than in mutants encoding wild-type *gdhR* in multiple laboratory strains with different genetic backgrounds. This study also provides a mechanism for this change in expression. GdhR P6S has limited binding to the *lctP* promoter compared to wild-type GdhR. The amino acid change is not in a predicted DNA-binding domain, and the mutation did not impact dimerization. However, the predicted GdhR structure suggests that P6S alters the secondary structure and flexibility of the DNA binding domain.

GISP also reports MICs for a panel of antibiotics, and the authors observed that isolates with *gdhR6* had higher azithromycin and ceftriaxone MICs than isolates encoding wild-type *gdhR.* However, the presence of *gdhR6* alone does not impact MICs in laboratory transformants. In clinical isolates, *gdhR6* is often found on genomic backgrounds with *mtrR-*P A-del, with 54% of isolates in the GISP panel with *gdhR6* also having *mtrR*-P A-del. The GISP isolates with a wild-type *mtrR* promoter did not have elevated MICs, demonstrating that *mtrR-*P A-del was responsible, at least in part, for this observation. Clinical isolates in the GISP panel had azithromycin MICs higher than would be expected if they only encoded the *mtrR-*P A-del, many above the defined susceptibility threshold (MIC > 1 μg/mL), suggesting that these isolates encode additional, resistance-associated alleles outside of the *mtr* operon or 23S rRNA loci.

The authors hypothesized that isolates with *mtrR-*P A-del acquire *gdhR6* to compensate for decreased expression of *lctP*. To test this hypothesis, they characterized *gdhR* and *mtrR–mtrR-*P alleles in a collection of whole-genome sequences from historical, Danish N. gonorrhoeae isolates (9), including isolates collected in the preantibiotic era and during the rise of resistance in the gonococcal population. The association between *gdhR6* and *mtrR*-P A-del was replicated in this data set. Unexpectedly, the authors found that *gdhR6* was in strains isolated in the preantibiotic era independent of selection for *mtrR-*P A-del. *gdhR6* did not influence the spontaneous mutation of the *mtrR* promoter in the laboratory. In isolates from the antibiotic era in this collection, the frequency of *gdhR6* rises and falls with the frequency of *mtrR-*P A-del, suggesting that the selection pressure of antibiotic use also influences the frequency of linked alleles and highlights the impact antimicrobial use has had on the gonococcal population.

Additional experimental and computational studies may illuminate the pressures contributing to the co-occurrence of *gdhR6* and *mtrR-*P A-del. The current work did not investigate whether isolates with both mutations represent a single, highly successful gonococcal lineage or multiple emergences of this combination. It is also unknown whether the preantibiotic era isolates with *gdhR6* were ancestors of current lineages carrying both *gdhR6* and *mtrR-*P A-del or if they are still circulating in the extant gonococcal population. In future work, phylogenetic analyses calibrated with collection dates could determine the order and timing in which these mutations are acquired and estimate the number of independent events. In addition to contributing to antibiotic resistance, mutations that cause overexpression of *mtrCDE* are also thought to be advantageous in the rectal environment, where the efflux pump mediates resistance to other hydrophobic compounds, including fecal lipids (10). The fitness of *gdhR6*, alone or in combination with *mtrR*-P A-del, under specific conditions or during infection of particular anatomical sites is a further avenue for future research.

In summary, Ayala et al. (6) discovered a variant in the repressor *gdhR* and defined its impact on the expression of the virulence factor *lctP* and linkage with resistance-associated alleles. This work underscores the importance of using diverse genetic backgrounds and combining experimental approaches with population-level analyses. These results support the need for future studies identifying loci contributing to gonococcal virulence, the interaction between virulence and antimicrobial resistance in N. gonorrhoeae, and the role of the genetic background on the acquisition and maintenance of antimicrobial resistance alleles.
